# Supplementary material for: Comparative multiomics analysis of cell physiological state after culture in a basket bioreactor
Source: Sci Rep. 2022 Nov 23;12:20161. doi: 10.1038/s41598-022-24687-4 (PMC9686226; doi:10.1038/s41598-022-24687-4)
Supplement: Supplementary file 1 — Supplementary Information 1. [file 41598_2022_24687_MOESM1_ESM.zip › raw data/Transcriptomic raw data/6.Enrichment/2.KEGG/TE1vsTC1/UP/src/csab04668.html]

csab04668


- csab:103226390

- Up regulated genes

103226390(2.68260263702081)

- csab:103236305

- Up regulated genes

103236305(1.72662479914397)

- csab:103240296

- Up regulated genes

103240296(1.18536997243945)

- csab:103222156

- Up regulated genes

103222156(1.83501644039466)

Close
